# Investigation of the Interaction Mechanism between Salbutamol and Human Serum Albumin by Multispectroscopic and Molecular Docking

**DOI:** 10.1155/2020/1693602

**Published:** 2020-02-10

**Authors:** Ting Zhao, Zihui Liu, Jingmei Niu, Baoxing Lv, Yuliang Xiao, Yuqin Li

**Affiliations:** ^1^Department of Clinical Pharmacy, Weifang People's Hospital, Weifang 261041, China; ^2^School of Chemical Science and Engineering, Tongji University, Shanghai 200092, China; ^3^School of Pharmaceutical Sciences, Shandong First Medical University and Shandong Academy of Medical Science, Taian 271016, China

## Abstract

Salbutamol (SBAL), a kind of short-acting beta 2-adrenergic agonist, has been mainly used to treat bronchial asthma and other allergic airway diseases clinically. In this study, the interaction mechanism between salbutamol and human serum albumin was researched by the multispectral method and molecular docking. The fluorescence intensity of HSA could be regularly enhanced with the increase of SBAL concentration. Both the results of the multispectral method and molecular docking showed that SBAL could bind HSA with van der Waals force and hydrogen bonds. The binding mechanism was further analysed by UV-Vis and synchronous fluorescence spectra. The contents of the secondary structure of free HSA and SBAL-HSA complex were evaluated using CD spectra.

## 1. Introduction

**Figure 1:**
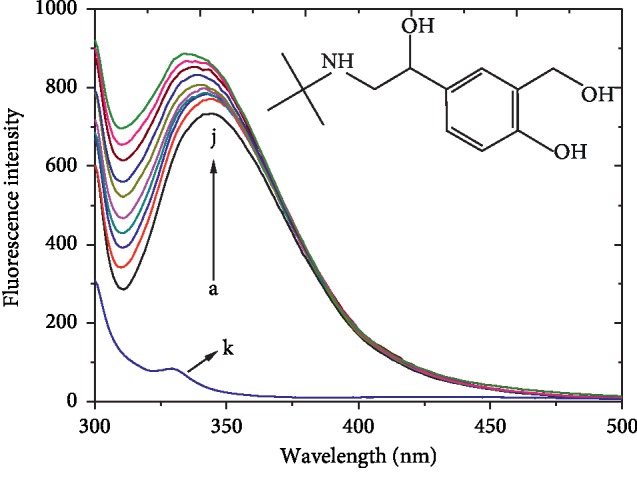
The chemical structure of SBAL and the SBAL-HSA fluorescence emission spectra. *C*_HSA_ = 1.5 *μ*Μ, whereas *C*_SBAL_ was 0, 3.33, 6.67, 10.0, 13.3, 16.7, 20, 23.3, 26.7, and 30.0 *μ*Μ from curves a to j, respectively. For the curve k, *C*_HSA_ = 0 *μ*Μ and *C*_SBAL_ = 10.0 *μ*Μ. Tris buffer, pH = 7.4, *T* = 288 K, and *λ*_ex_ = 295 nm.

Salbutamol (SBAL, Figure 1), a short-acting *β*2-adrenergic receptor agonist, has been basically used to treat bronchial asthma and other allergic airway diseases clinically [1, 2]. Moreover, SBAL also has been used to treat acute hyperkalemia, cystic fibrosis, and spinal muscular atrophy and relax the uterine smooth muscle to delay premature labor [3]. SBAL has been confirmed to burn fat and improve muscle weight in rats, so it was abused as a growth promoter and lipid-lowering agent reducing fat deposition in cattle, sheep, pigs, and poultry [4, 5]. And it was also used as an illegal drug to improve athletes' muscles to achieve good results [6, 7]. The most common side effects of SBAL are fine tremor, anxiety, headache, muscle cramps, dry mouth, and palpitation, and high doses might cause hypokalaemia [8]. Meat products containing residual salbutamol could cause great harm to the human body [9]. So, SBAL has been forbidden as a lipid-lowering agent in meat-producing animals in many countries [10, 11]. And since it is an illegal drug, athletes are not allowed to take it [12, 13].

Human serum albumin (HSA) accounts for about 60% of the total plasma protein and is a common model protein used to study drug-protein interactions. HSA shows a high affinity to different materials including drugs, poisons, nutrients, metal ions, and their metabolites [14–16]. Based on materials' affinity to HSA, their absorption, distribution, metabolism, and toxicity could be changed in vivo and then affect their pharmacokinetics, pharmacodynamics, and toxicity. In general, the weak binding drug with HSA could result in a short eliminate time or undesirable distribution, whereas the strong one could raise the drug concentrations in human plasma. HSA could also maintain the blood pH and colloid osmotic blood pressure [17]. To guarantee the safety and efficacy of drug, the binding rate of drug-protein must be first developed in designing a new drug.

In this paper, the interaction mechanism between SBAL and HSA was researched at three different temperatures using fluorescence, synchronous fluorescence, ultraviolet-visible (UV-Vis), and circular dichroism (CD) spectrometry under the simulated physiological conditions; meanwhile, molecular docking was used to investigate the binding sites.

## 2. Materials and Methods

### 2.1. Materials

Human serum albumin (HSA, content was 96–99%), obtained from Sigma Chemical Company in China, was directly used in the experiment with no further purification and the Mw was specified as 66,500 Da. HSA stock solution (15 *μ*M) was made in the 0.05mM Tris-HCl buffer solution (pH 7.4 ± 0.1) and placed in the dark at 0∼4°C. SBAL was presented by Jinwei Pharmaceutical (Shandong) Co., Ltd. and the 1.0 mM stock solution was made in absolute methanol. 50 mM (pH 7.4 ± 0.1) Tris-HCl buffer solution containing 0.10 M NaCl was used to maintain the pH and ionic strength of all solutions. The remaining reagents were of analytical grade. Wahaha pure water was used for all experiments.

### 2.2. Methods and Apparatus

#### 2.2.1. Measurement of Fluorescence Spectra

First, 0.5 mL HSA stock solution was placed in a 5.0 mL volumetric flask, and then SBAL stock solution was sequentially added in it and diluted to 5.0 mL with the Tris-HCl buffer. Finally, a series of SBAL concentration work solutions containing HSA 1.5 *μ*M were incubated for 5 min at 288 K, respectively. The fluorescence spectra were recorded at 300∼500 nm by setting the Trp214 excitation wavelength to 295 nm by an F-4500 fluorescence spectrometer (Hitachi, Japan) and by fixing the excitation and emission slits to 5 nm. According to the above method, the effect of temperature on the interaction of SBAL-HSA complex was estimated at 288, 300, and 310 K when the excitation/emission wavelength was set at 295/344 nm.

The synchronous fluorescence spectra of HSA containing different concentrations of SBAL were measured when the difference between the emission and excitation wavelengths was installed at 15 and 60 nm, respectively. The HSA concentration in all the sample solutions was 1.5 *μ*M.

#### 2.2.2. Measurement of Absorption Spectra

The UV absorption spectra of HSA free and in the presence of SBAL were measured in the range of 200–400 nm using a UV-2450PC (Shimadzu, Japan) by 1.0 cm quartz cells. All the work solutions contained 1.5 *μ*M HSA and were incubated for 5 min at 288 K.

#### 2.2.3. Measurement of CD Spectra

The CD spectra of HSA free and in the presence of SBAL were measured on a JASCO J-810 (Japan) using a 0.1 cm quartz cell at 288 K. The buffer solution as the blank was used to correct the CD spectra. The difference CD spectra of HSA and SBAL-HSA were obtained automatically by subtracting the blank after finishing scan. To obtain the average CD spectra, each sample was tested three times. The secondary structure content of HSA was estimated using SELCON3 program.

#### 2.2.4. Molecular Docking

SBAL docking with HSA was performed by MOE2009 (Chemical Computing Group Inc., Montreal, Canada). The structure of SBAL was retrieved from DrugBank (http://www.drugbank.ca). Its 3D structures were made with SYBYL-X (Tripos Inc., St. Louis, USA). The optimal geometry conformation of SBAL was done by the distance-dependent dielectric function in the standard Tripos force field [18, 19] with the energy gradient of 0.001 kcal mol^−1^. Gasteiger–Hückel charges were applied to the ligand atoms. The crystal structure of HSA combined with warfarin was selected from the Brookhaven Protein Data Bank and used for docking. First, the hydrogen atom was added to the HSA structure. Then, the complex of HSA-SBAL was manipulated to content the requisition of docking. The lowest energy and *S* value of the conformation were specified as the final evaluation standard.

## 3. Results and Discussion

### 3.1. Effect of SBAL on the Intrinsic Fluorescence of HSA

Tryptophan (Trp) residue, phenylalanine (Phe) residue, and tyrosine (Tyr) residue are the fluorescence groups in HSA. But the quantum yield of Phe residue is very low, and the fluorescence intensity of Tyr residue is almost completely quenched when it is ionized or close to an amino group, a carboxyl group, or a Trp. So, the intrinsic fluorescence of HSA mainly comes from only one Trp214 [20]. The Trp214 is located in subdomain IIA of Sudlow's binding site I and gave the strongest emission at about 340 nm in the fluorescence spectrum of HSA when the excitation wavelength is 295 nm. The fluorescence intensity of Trp-214 is aeschynomenous to the surroundings and it would be easily affected even if the microenvironment of HSA was changed a little, such as ligand interaction, the conformational change, and denaturation of HSA.

Figure 1 shows the fluorescence spectra of HSA free and in the presence of SBAL by setting an excitation at 295 nm. In Figure 1, the curves a and k show the fluorescence spectrum of free HSA and free SBAL and the curves b–j show fluorescence spectra of SBAL-HSA, respectively. It was found that free SBAL had no distinct fluorescence, and the continuous increase in the concentration of SBAL resulted in a regular increase in the fluorescence intensity of HSA at 344 nm, while the maximum emission wavelength shifted from 344 to 336 nm. The results signified that the binding site of SBAL was near the Trp214 in HSA and the Trp214 microenvironment was altered to more hydrophobic [21]. The effect of SBAL on the fluorescence intensity of HSA is similar to the interaction between syringin and HSA [20].

### 3.2. Measurement of Binding Constants

To further illuminate the interaction mechanism of SBAL-HSA, the Bhattacharya equation (equation (1)) [19, 22] was used to process the fluorescence intensity value (*λ*_ex_\*λ*_em_, 295\344 nm) at three temperatures 288, 300, and 310 K:

(1) $$\begin{array}{c} \frac{1}{\Delta F}=\frac{1}{\Delta F_{\text{max}}}+\frac{\Delta F_{\text{max}}}{K\left[Q\right]}. \end{array}$$

**Figure 2:**
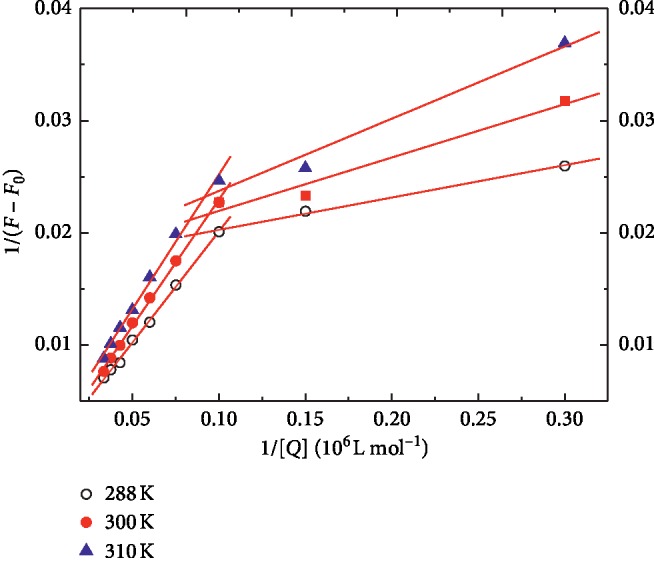
Plot of 1/(*F*_*x*_ − *F*_0_) against 1/[*Q*] at three different temperatures. *λ*_ex_ = 295 nm, *λ*_em_ = 344 nm, Tris buffer, and pH = 7.4.

In equation (1), Δ*F* = *F*_*x*_ − *F*_0_ and Δ*F*_max_ = *F*_∞_ − *F*_0_. *F*_0_, *F*_*x*_, and *F*_∞_ express the fluorescence intensities of free HSA and at some intermediate and saturation molar concentration of SBAL, respectively. *K* represents the binding constant and [*Q*] represents the SBAL molar concentration.  Figure 2 records the Bhattacharya curves of the SBAL-HSA at 288, 300, and 310 K, respectively. In Figure 2, all the plots for the HSA-SBAL had two regression curves, which signified that SBAL bonded to HSA in two types of binding sites with the SBAL critical concentration of 0.1 *μ*M. The binding constant of the first type was named *K*_1_ when the SBAL concentration was less than 0.1 *μ*M, and *K*_2_ was the binding constant of the second type when the SBAL concentration was greater than 0.1 *μ*M. From Table 1, *K*_1_ was about 10 times *K*_2_, and the results suggested that the first binding site had higher affinity and selectivity than the second with the lower bond energy. Meanwhile, the value of *K*_1_ and *K*_2_ decreased with the increase in temperature that implied that a stable complex might be formed between SBAL and HSA, and it might be decomposed partially with the temperature increase.

### 3.3. Measurement of Thermodynamic Parameters and the Binding Forces

There are four types of noncovalent bond interactions between ligand and protein, including electrostatic interactions, hydrogen bonds, hydrophobic interactions, and van der Waals forces. Thermodynamic parameters include free energy (Δ*G*°), standard enthalpy (Δ*H*°), and standard entropy (Δ*S*°), which Δ*G*° can be used as the criterion of spontaneous reaction under constant temperature and pressure. The signs and magnitudes of Δ*G*°, Δ*H*°, and Δ*S*° are crucial in any reaction and can be used to estimate the types of interaction forces in the formation of SBAL-HSA complex [23]. The relationship between binding constants and thermodynamic parameters of chemical reactions could be described by the following equations [23]:

(2) $$\begin{array}{c} \ln\;K=-\frac{\Delta H^{0}}{RT}+\frac{\Delta S^{0}}{R}, \end{array}$$

(3) $$\begin{array}{c} \Delta G^{0}=\Delta H^{0}-T\Delta S^{0}. \end{array}$$

In (2), *R* represents the gas constant. Based on the binding constant *K* from Table 1, thermodynamic parameters were calculated using equations (2) and (3). The Δ*H*°, Δ*G*°, and Δ*S*° at three different temperatures are also listed in Table 1 and they were all negative. Δ*G*° < 0 demonstrated that SBAL bonded spontaneously with HSA. Both negative values of Δ*H*° and Δ*S*° represented a typical van der Waals force and hydrogen bonds were involved in the formation of SBAL-HSA complex.

### 3.4. Estimation of Energy Transfer between SBAL and HSA

**Figure 3:**
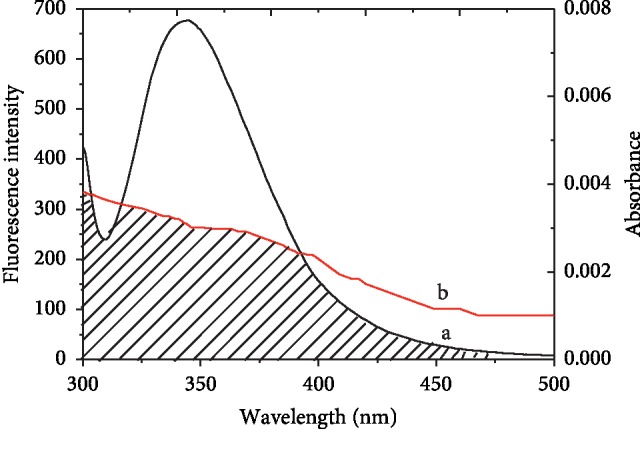
The overlap of (a) the absorption spectra of SBAL and (b) the fluorescence emission spectrum of HSA. *C*_HSA_ = 1.5 *μ*Μ and *C*_SBAL_ = 1.5 *μ*Μ (288 K pH = 7.4, *λ*_ex_ = 295 nm).

Nonradiation energy transfer (NRET) results could offer a lot of information on ligand-protein. The rate of energy transfer is related to the overlap extent between the protein's fluorescence spectrum and the ligand's absorbance spectrum, the relative orientations of protein and ligand diploes, and the distance (*r*) between protein and ligand.  Figure 3 presents the fluorescence emission spectrum of HSA overlapped with the absorption spectrum of SBAL. NRET follows the following equations:

(4) $$\begin{array}{c} E=\frac{R_{0}^{6}}{R_{0}^{6}+r^{6}}, \end{array}$$

(5) $$\begin{array}{c} E=1-\frac{F}{F_{0}}, \end{array}$$

(6) $$\begin{array}{c} R_{0}^{6}=8.8\times 10^{-25}K^{2}N^{-4}\phi J, \end{array}$$

where *E* represents the energy transfer efficiency and *R*_0_ represents the critical transfer distance of the 50% transfer efficiency [17]. *F*_0_ and *F* represent the fluorescence intensity of free HSA or SBAL-HSA, respectively. About HSA, *K*^2^ = 2/3, *ϕ* = 0.118, and *N* = 1.336 [17]. *J* represents the overlap integral value in Figure 3. *J* could be estimated using the following equation:

(7) $$\begin{array}{c} J=\frac{\sum \left\{F\left(\lambda \right)\varepsilon \left(\lambda \right)\lambda^{4}\Delta \lambda \right\}}{\sum \left\{F\left(\lambda \right)\Delta \lambda \right\}}. \end{array}$$

In equation (7), *F*(*λ*) expresses the HSA fluorescence intensity at some *λ*_em_, *ε*(*λ*) expresses the SBAL molar absorption coefficient (cm^−1^ mol^−1^) at the same *λ*_em_ [24]. Herein, *J* and *E* were equal to 9.68 × 10^−15^ cm^3^ M^−1^ and 0.10, respectively. So, *R*_0_ and *r* were equal to 2.50 and 1.96 nm, respectively. *r* < 7 nm and 0.5*R*_0_ < *r* < 1.5 *R*_0_ obeyed the criteria [25]. The results complied that the energy transfer existed between SBAL and HSA. Therefore, it is indicated that SBAL as a sensitizer could offer a suggestion to its location near Trp214 in HSA.

### 3.5. Effect of SBAL on the HSA Conformation

Synchronous fluorescence, UV-Vis, and CD spectroscopic methods were used for further illustration of the interaction mechanism of SBAL with HSA and the content change of HSA secondary structure after binding interaction.

#### 3.5.1. Effect of SBAL on the HSA Synchronous Fluorescence Spectra

Synchronous fluorescence spectrum could show the fluorescence change and the shift of fluorescence emission wavelength, which is due to the polarity alteration of the chromophore microenvironment. The synchronous fluorescence spectrum was obtained by simultaneously scanning the excitation and emission monochromators while setting a constant wavelength difference between them to get information in the microenvironment for Tyr (Δ*λ* = 15) or Trp (Δ*λ* = 60) [26].

**Figure 4:**
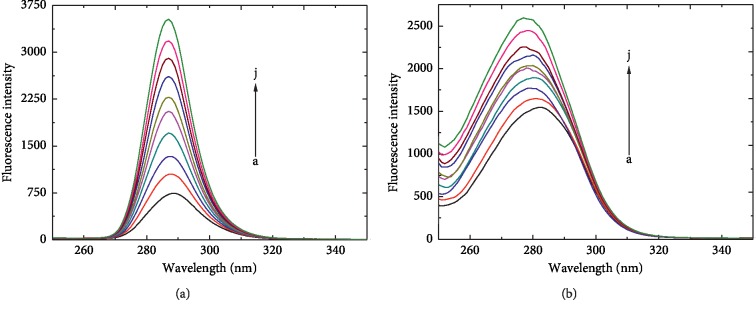
The SBAL-HSA synchronous spectra (a) Δ*λ* = 15 nm and (b) Δ*λ* = 60 nm of the patulin-HSA system. *C*_HSA_ = 1.5 *μ*Μ, and other conditions were the same as in Figure 1.

The synchronous fluorescence spectra of the SBAL-HSA system are shown in Figure 4. It was found in Figure 4(a) that the fluorescence intensity of HSA increased with the increase in SBAL concentration and the fluorescence emission wavelength did not shift obviously, illustrating that the microenvironment of Tyr residues maintained unchanged. On the contrary, the fluorescence intensity of HSA not only increased with the increase in SBAL concentration and the fluorescence emission peak was blue-shifted from 282 to 276 nm obviously, indicating an alternated microenvironment around Trp214 in Figure 4(b). It was implied that the conformation of HSA was changed with SBAL binding and resulted in such a manner that the polarity around Trp-214 was decreased markedly. Therefore, the results illustrated that the binding site of SBAL with HSA was near the Trp214. The microenvironment polarity was lowered, and the hydrophobicity was heightened around the Trp214 residues due to the hydrophobic part of SBAL approached enough to the phenyl of Trp214.

#### 3.5.2. Effect of SBAL on the HSA UV-Vis Spectra

**Figure 5:**
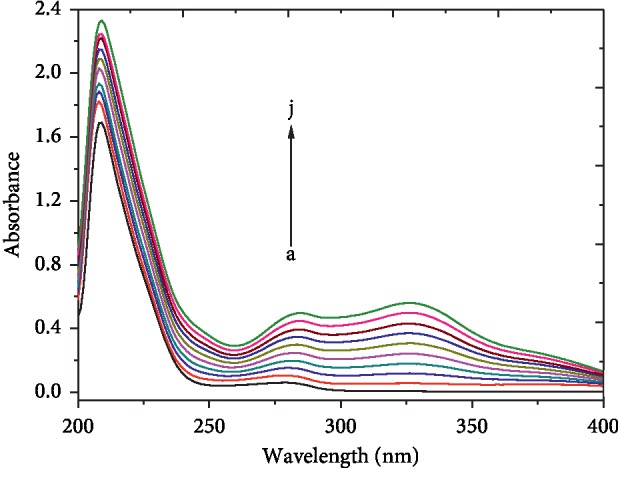
UV absorption spectra of the SBAL-HSA system. *C*_HSA_ = 1.5 *μ*Μ, and other conditions were the same as in Figure 1.

The HSA UV-Vis absorption spectra with and without SBAL are plotted in Figure 5. From Figure 5, two absorption peaks of free HSA at 209 and 278 nm were observed, which presented the conformations of HSA backbone and aromatic amino acids, respectively. With SBAL binding, the peak at 209 nm was blue-shifted about 1 nm along with an increase in absorbance intensity, which indicated the microenvironment of Trp214 became more hydrophobic. Meanwhile, the peak at 278 nm was red-shifted about 7 nm along with an absorbance intensity increase because SBAL had a stronger absorbance peak at 276 nm. The new peak occurred at 328 nm might due to the complex of SBAL-HSA.

#### 3.5.3. Effect of SBAL on the HSA CD Spectra

**Figure 6:**
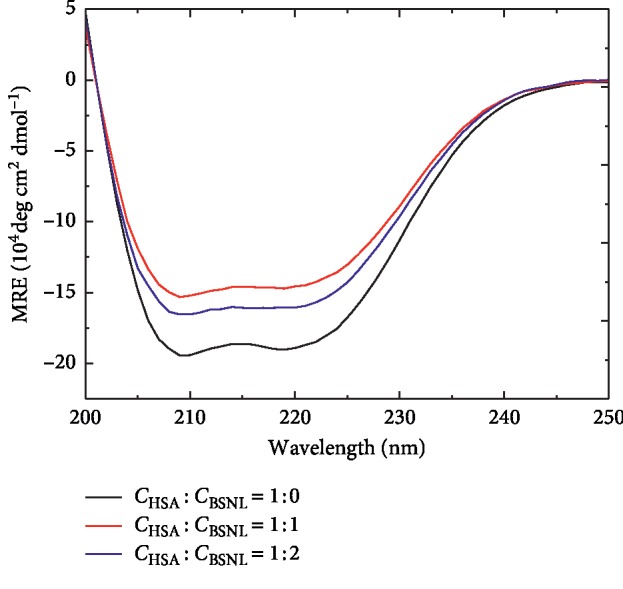
The SBAL-HSA CD spectra. *C*_HSA_ = 1.5 *μ*Μ, pH = 7.4, *T* = 288 K.

CD is a common method to monitor the secondary structure alternation of HSA by a ligand binding. The CD graphs of HSA either in the existence or nonexistence of SBAL are plotted in Figure 6, and the secondary structure contents of HSA were estimated using SELCON3 program which is summarized in Table 2. In Figure 6, the free HSA CD spectra show two (209 and 219 nm) conspicuous negative bands, which responded to the HSA *α*-helical structure [27]. The band_209_ and band_219_ intensities of HSA increased with the molar concentration ratio of HSA and SBAL increasing from 1 : 0 to 1 : 1, while the *α*-helix content of HSA decreased from 56.0% to 48.5% along with *β*-sheet content improved from 3.8% to 10.3% and the *β*-turn (20.4 to 20.5%) and the random coil (19.8 to 20.7%) content little changed. But the intensity of the HSA band at 209 and 219 nm decreased with the molar concentration ratio of HSA and SBAL further increasing from 1 : 1 to 1 : 2, while the *α*-helix contents of HSA increased from 48.5% to 52.6% along with the random coil content of the HSA decreased from 20.7% to 16.9% and the *β*-sheet (10.3 to 11.3%) and the *β*-turn (20.5 to 19.2%) content little changed. Therefore, the CD results further confirmed that SBAL bonded to HSA with two binding sites and were in line with the binding constant consequence.

### 3.6. Molecular Docking

**Figure 7:**
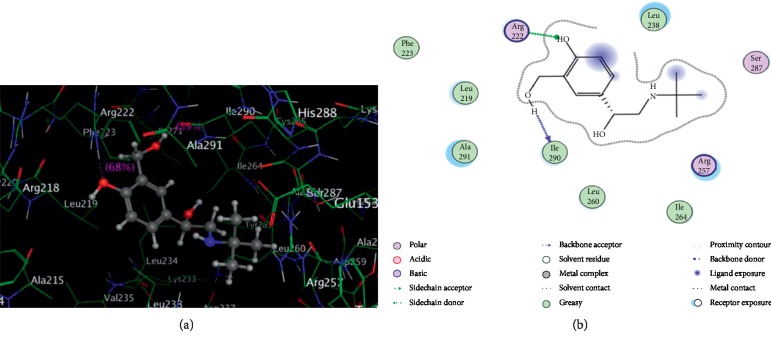
The SBAL-HSA molecule docking (a) and a projection of Figure 7(a) (b). The amino acid residues in HSA are shown by lines, and SBAL structure is shown by a ball-and-stick formula. Hydrogen bonds in SBAL-HSA are shown by dotted line.

There are three similar structure domains (I–III): I (1–195 residue) and II (196–383 residues) in HSA crystal structure and each one is composed of 6A and 4B substructure domains, which form a barrel-shaped hydrophobic cavitary [28]. The binding site of ligand to HSA is mainly located in the hydrophobic gap of IIA (site I) and IIIA (site II). Trp214 in HSA situates in subdomain IIA. In order to probe the SBAL binding site, HSA-warfarin complex was selected as a model [29] and HSA-SBAL docking study has been done. The most reasonable combining way of SBAL-HSA is presented in Figure 7, where only residues in the 6 Ǻ range around SBAL are shown, and the residues involved in the binding of SBAL within the active site of HSA are listed in Table 3. From Figures 7(a) and 7(b) and Table 3, SBAL was inserted into the barrel-shaped structure of subdomain IIA (residues 222–291). Leu219, Phe223, Leu238, Leu264, and Ile264 residues formed a hydrophobic cavity and the nonpolar inside wall of the cavity bonds to alkalescent SBAL with van der Waals force. Alkaline Arg222 residues bond to phenol hydroxyl of SBAL by hydrogen bond, and Ile290 as an acceptor formed a hydrogen bond with the sidechain hydroxyl of SBAL. The results of molecular docking showed that SBAL could bind HSA with van der Waals force and hydrogen bonds, which was corresponded to “3.3” and the reference results in [30]. The calculated binding Δ*G*° was −11.81 kJ mol^−1^ K^−1^, which is close to the experimental value (−18.18 kJ mol^−1^ K^−1^, 288 K) to a certain degree.

## 4. Conclusion

In the study, the interaction mechanism between SBAL and HSA was researched under a simulated physiological condition using a combination of experimental methods and molecular docking. The binding mechanism of SBLA-HSA was determined using fluorescence spectroscopy, with measuring changes of the Trp214 fluorescence. Synchronous fluorescence, UV-Vis, and CD spectroscopic methods were used to study the secondary structures and interactions between SBAL and HSA. On the above findings, the binding models were built by molecular docking, thus providing the involved residue information about the complex formation. SBAL was found to be located at Sudlow site I near Trp214. The results suggested van der Waals force and hydrogen bonds played an important role in the binding of SBAL-HSA.

## Figures and Tables

**Table 1:** Binding constants and thermodynamic parameters of SBAL-HSA system at different temperatures.

T (K)	*K* (L mol^−1^)		Δ*G*° (kJ·mol^−1^ K^−1^)	Δ*S*° (J·mol^−1^)	Δ*H*° (kJ·mol^−1^)
288	*K*_*1*_ × 10^4^	1.6121 ± 0.71	−23.52 ± 0.85	−93.53 ± 0.82	−50.46 ± 0.76
300		1.0886 ± 0.58	−22.40 ± 0.92		
310		0.3472 ± 0.81	−21.47 ± 0.73		

288	*K*_*2*_ × 10^3^	2.0094 ± 0.44	−18.18 ± 0.25	−31.41 ± 0.37	−27.23 ± 0.29
300		1.2214 ± 0.50	−17.80 ± 0.32		
310		0.9001 ± 0.78	−17.49 ± 0.54		

**Table 2:** Effect of the different SBAL concentrations on the secondary structure content of HSA.

*C*_HSA_ : *C*_BSAL_	*α*-Helix (%)	*β*-Strand (%)	*β*-Turns (%)	Unordered (%)
1 : 0	56.0	3.8	20.4	19.8
1 : 1	48.5	10.3	20.5	20.7
1 : 2	52.6	11.3	19.2	16.9

**Table 3:** The residues were involved in the binding of SBAL within the active site of HSA.

Complex	Δ*G*° (kcal mol^−1^)	The involved residues	
		Hydrogen bond	van der Waals force
SBAL-HSA	−11.81	Arg222, le290	Leu219, Phe223, Leu238, Leu260, Ile264, Ile290, Ala291

## Data Availability

The data used to support the findings of this study are included within the article.

## Abbreviations

SBAL: Salbutamol
HSA: Human serum albumin
CD: Circular dichroism
*λ*_ex_: Excitation wavelength
*λ*_em_: Emission wavelength
Trp: Tryptophan
Tyr: Tyrosine
Phe: Phenylalanine.
